# Asymmetric cell division of granule neuron progenitors in the external granule layer of the mouse cerebellum

**DOI:** 10.1242/bio.009886

**Published:** 2015-05-15

**Authors:** Parthiv Haldipur, Iswariya Sivaprakasam, Vinod Periasamy, Subashika Govindan, Shyamala Mani

**Affiliations:** 1 National Brain Research Centre, Manesar, Gurgaon 122050, Haryana, India; 2 Centre for Neuroscience, Indian Institute of Science, Bangalore 560012, Karnataka, India

**Keywords:** Cerebellum, Cell division, β-Catenin, Sonic hedgehog

## Abstract

The plane of division of granule neuron progenitors (GNPs) was analysed with respect to the pial surface in P0 to P14 cerebellum and the results showed that there was a significant bias towards the plane of cell division being parallel to pial surface across this developmental window. In addition, the distribution of β-Catenin in anaphase cells was analysed, which showed that there was a significant asymmetry in the distribution of β-Catenin in dividing GNPs. Further, inhibition of Sonic Hedgehog (Shh) signalling had an effect on plane of cell division. Asymmetric distribution of β-Catenin was shown to occur towards the source of a localized extracellular cue.

## INTRODUCTION

A key constituent of the central nervous system of all jawed vertebrates is the cerebellum (Chaplin et al., 2010). The stereotypical circuit of the cerebellum involves limited defined cell types that have been well characterized for the past 100 years. Of the cells that make up the circuitry of the cerebellum, the granule neurons (GNs) are the most numerous and comprise the single largest neuronal type in the central nervous system (Fox and Barnard, 1957). The differentiation of GNs of the cerebellum is unique. GNPs arise from the rhombic lip and migrate tangentially to cover the surface of the cerebellum forming the external granule layer (EGL) (Miale and Sidman, 1961; Alder et al., 1996). During the first two postnatal weeks in the mouse, GNPs in the EGL initially proliferate and subsequently become postmitotic within the EGL (Altman, 1972). Postmitotic neurons migrate tangentially within the EGL first and then inwards on Bergmann glial fibers to form the mature GNs of the internal granule layer (IGL) (Komuro et al., 2001).

The role of the plane of cleavage during cell division and its consequence for cell fate has been investigated in several model organisms. In *Drosophila melanogaster*, neuroectodermal cells that divide parallel to the axis of apical-basal polarity results in both daughter cells retaining their neuroectodermal identity. In contrast, when the plane of division is rotated 90 degrees and is perpendicular to apical-basal polarity, the basal daughter cell becomes the neuroblast and delaminates, whereas the apical cell remains in the neuroectodermal layer (Wodarz and Huttner, 2003). Work from several groups have led to our understanding of heterogeneity of progenitor cell populations and how they divide to give rise to the differentiated neurons of the cerebral cortex (Chenn and McConnell, 1995; Fishell and Kriegstein, 2003; Costa et al., 2008; Fish et al., 2008; Knoblich, 2008). Several of these studies have analysed the orientation of cell division with respect to the ventricular zone (VZ) of the developing cerebral cortex (Chenn et al., 1998; Haydar et al., 2003; Sanada and Tsai, 2005). The apical membrane of the apical progenitors abuts the lumen of the lateral ventricle and the orientation of the cleavage plane with respect to the ventricular zone results in either both daughter cells inheriting the apical membrane, a symmetric division, or only one daughter cell inheriting the apical membrane, an asymmetric division. Because the apical membrane is narrow, a slight tilt in the axis of cell division from vertical would result in an asymmetric versus a symmetric division (Kosodo et al., 2004). In contrast, basal progenitors of the subventricular zone which lack apical-basal polarity display randomly oriented cleavage planes (Attardo et al., 2008; Noctor et al., 2008). Mutations that affect the accuracy of spindle pole alignment and shift the plane of cell division in apical progenitors have drastic consequences for cell fate. One example is primary microcephaly, a recessive autosomal disorder in which the brain size is greatly reduced. To date almost all disease causing mutations have been mapped to genes that code for proteins that localize to the centrosome at some point in the cell cycle (Kaindl et al., 2010; Farag et al., 2013). One such example is Abnormal Spindle-like, Microcephaly-associated (ASPM), which when mutated results in disruption of cleavage plane, and depletion of progenitor cells because of asymmetric localization of fate determinants that ultimately leads to a small brain (Knoblich, 2008). While the studies described above indicate a trend with respect to plane of division in the VZ of the cerebral cortex, we do not know whether such a mechanism exists in secondary zones of proliferation such as the EGL of the developing cerebellum. Therefore, whether the orientation of cell division with respect to the pial surface (closest to the proliferating GNP and opposite to the direction of migration as in the case of neuronal progenitors of the VZ) was random or whether there was a bias in the orientation of plane of cell division was analysed. GNP proliferation has been shown to play a role in increasing the surface area and foliation of the cerebellum (Sudarov and Joyner, 2007) and Shh signalling is a regulator of GNP proliferation (Wechsler-Reya and Scott, 1999). Therefore, Shh signalling *in vivo* was perturbed to see if this would be accompanied by changes in the plane of cell division in GNPs.

The role of Wnt has been studied in the context of early patterning of the hindbrain region (McMahon and Bradley, 1990). However, more recent studies have looked at the effect of Wnt signalling pathway on cerebellar development (Schüller and Rowitch, 2007). Activation of β-Catenin did not seem to affect the formation of the EGL, however it resulted in a defect in proliferation and survival of GNPs leading to a hypoplasic cerebellum (Pei et al., 2012; Pöschl et al., 2013). Further, constitutive activation of Wnt-β-Catenin signalling resulted in premature differentiation of GNPs (Lorenz et al., 2011). Non-canonical Wnt signalling pathway can act to antagonize Shh signalling leading to the differentiation of GNPs (Anne et al., 2013). In the cerebellum, while mutations in the Wnt signalling pathway that includes β-Catenin can lead to medulloblastomas, this has been attributed to disrupted proliferation of neural stem cells from the lower rhombic lip and not from the region that gives rise to the EGL (Gibson et al., 2010). In the VZ, β-Catenin is an integral component of the Wnt signalling pathway that along with other molecules such as prominin1, par3, aPKC is part of the apically located adherens junction (Fietz and Huttner, 2011). Overexpression of β-Catenin leads to an increase in the number of cortical neuron progenitors and a subsequent expansion of cortical surface area in mice (Chenn and Walsh, 2002) and elimination of β-Catenin from neural progenitors *in vivo* causes premature neuronal differentiation (Woodhead et al., 2006). Given the role of β-Catenin in progenitor cells, its distribution between two daughter cells was analysed during GNP cell division.

## RESULTS

### Plane of cell division of GNPs in the EGL at different developmental ages

**Fig. 1.**
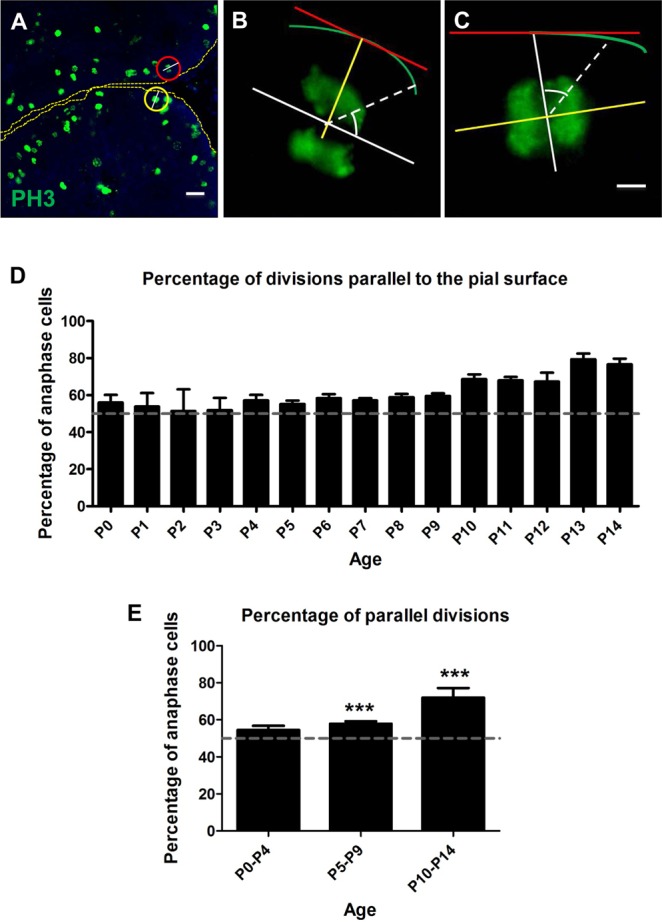
**The age-wise distribution of cell divisions that are parallel and perpendicular to the plane of the pia.** (A) Phosphohistone H3 (PH3) immunohistochemistry (green) on P6 mouse cerebellum. Divisions were classified into parallel (red circle) and perpendicular (yellow circle). (B) Magnified view of a cell whose plane of division (white line) is parallel to the pial surface (red line). (C) Magnified view of a cell whose plane of division (white line) is perpendicular to the pial surface (red line). (D) Weighted mean and standard deviations of percentage of parallel cell divisions plotted for each age. (E) Age-wise grouped weighted mean and standard deviations of percentage of parallel cell divisions. The percentage of parallel divisions increases significantly between P0–P4 and P5–P9 and also between P0–P4 and P10–P14 (***p<0.001); Unpaired two tailed *t*-test (n>5). The grey line in both graphs is drawn at 50% which would be the percentage of obtaining a parallel division if there was no bias. Error bars in D and E represent standard deviation.

EGL of the developing cerebellum was analysed to see whether the perpendicular and parallel orientations of cell division were equal and remain the same across ages. Cells were identified in anaphase using PH3 immunohistochemistry (Fig. 1A-C). Results indicate that between P0 and P4, the percentage of cell divisions parallel to the plane of the pial surface versus divisions perpendicular to the plane of the pial surface, remained around 50%. However, the number of parallel divisions increased gradually between P5–P14 (Fig. 1D,E).

### Perturbation of Sonic hedgehog signaling results in shifts in the plane of cell division

**Fig. 2.**
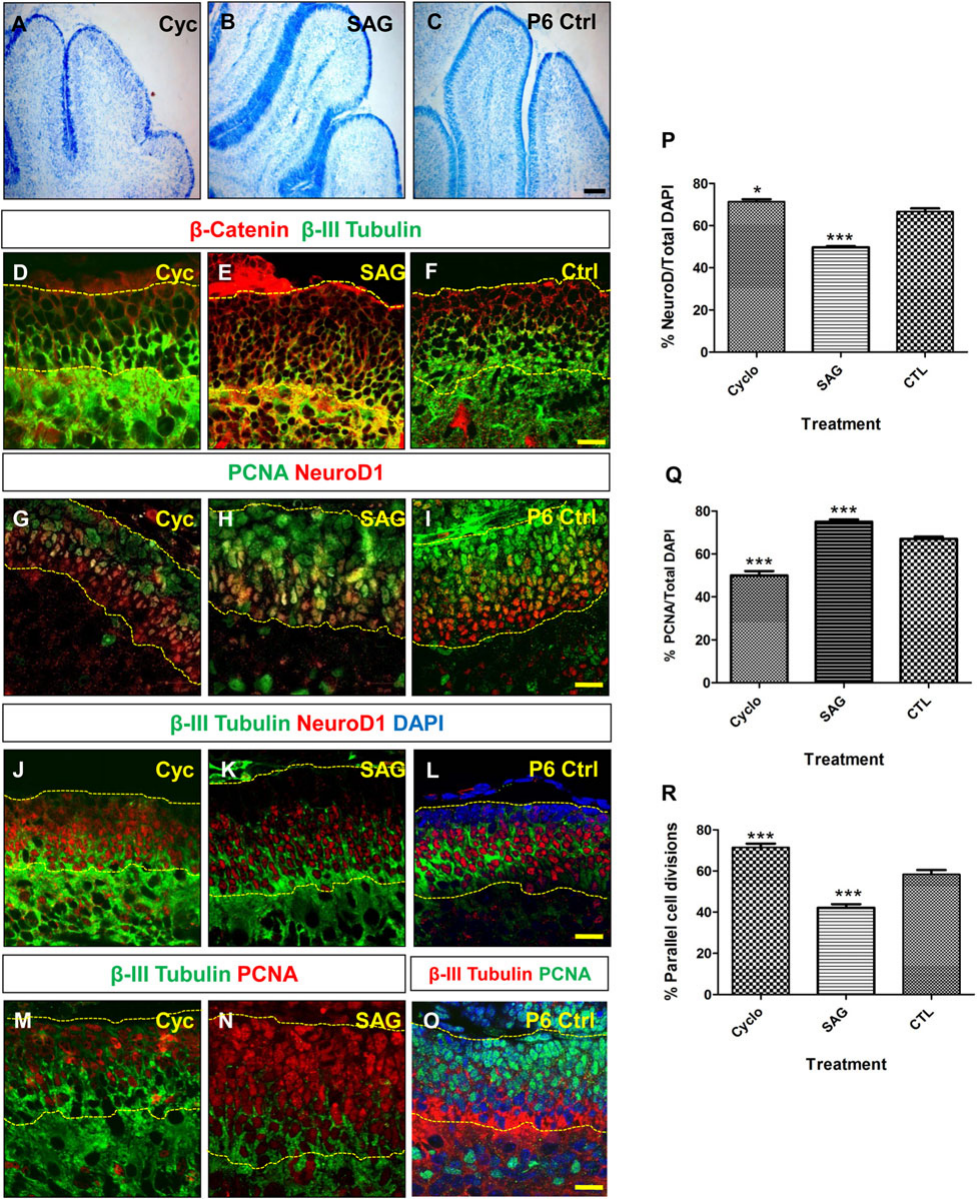
**Perturbation of Shh signalling induces a change in the level of neurogenesis in the cerebellar EGL** (A-C) Nissl staining of the Cyclopamine treated (Cyc), SAG treated, and P6 control (Ctrl) animals respectively showing the thickness of the EGL. Scale bar=100 µm. (D-F) β-Catenin and β-III Tubulin immunohistochemistry in Cyclopamine, SAG treated and control animals. (G-I) PCNA, NeuroD1 immunohistochemistry in Cyclopamine, SAG treated and control animals. (J-L) β-III Tubulin, NeuroD1 immunohistochemistry in the P6 cerebellum from Cyclopamine and SAG treated and control animals. (M-O) PCNA, β-III Tubulin immunohistochemistry in Cyclopamine and SAG treated and control animals. The yellow dotted line in Figure 2D-O represents the boundaries of the external granule layer (EGL). Scale bar=20 µm in D-O. (P) Graph showing an increase in the percentage of NeuroD1^+^ cells in Cyclopamine treated animals as compared to control (*p<0.05). The percentage of NeuroD1^+^ cells is significantly lower in the SAG treated animal as compared to P6 control (***p<0.001) (*n=*3, where *n=*number of animals). Data represented as mean±s.e.m percentage of NeuroD1 positive cells. (Q) Graph showing a decrease in the percentage of PCNA positive cells in Cyclopamine treated animals as compared to control (***p<0.001). The percentage of PCNA^+^ cells is significantly higher in the SAG treated animal as compared to P6 control (*p<*0.001) (*n=*3). Data represented as mean±s.e.m percentage of PCNA positive cells. (R) Graph showing the percentage of parallel (horizontal) cell divisions in Cyclopamine and SAG treated animals and P6 controls (*n*>3) The number of parallel cell divisions is significantly higher in the EGL of Cyclopamine treated mice (***p<0.001). SAG treatment on the other hand significantly reduces the number of horizontal divisions in the cerebellar EGL (***p<0.001). Data represented as mean±s.e.m. percentage of parallel cell divisions. All *p* values are based on student *t*-test.

Sonic hedgehog (Shh) regulates GNP proliferation (Wechsler-Reya and Scott, 1999). Therefore, the effect of the perturbation of Shh signaling on GNP cell division was investigated. Cyclopamine was used to inhibit Shh signaling and SAG was used to increase Shh signaling. P0 pups were treated with either Cyclopamine, or SAG for 6 days, and sacrificed on postnatal day 6. The EGL in Cyclopamine treated animals was thinner than the control, while SAG treated animals had a thicker EGL (compare Fig. 2J,K with Fig. 2L). The expression of β-Catenin in Cyclopamine treated animals was reduced and expanded in SAG treated animals (Fig. 2D-F). Cyclopamine treatment resulted in an overall increase in NeuroD1 positive cells (72%), as compared to control animals (65%), while SAG treated animals showed a decrease in NeuroD1 positive cells (45%). In both conditions, the difference was statistically significant (Fig. 2G-L,P; graph). There was also a significant decrease in PCNA positive proliferative cells in Cyclopamine treated animals (50%), as compared to control (66%) and SAG treated animals (75%). SAG treated animals showed a significantly higher percentage of PCNA positive cells as compared to control animals (Fig. 2G-I,Q; graph). The region of β-III Tubulin expression was also expanded into the outer EGL in Cyclopamine treated mice, while its expression domain was reduced in SAG treated mice (Fig. 2J-O). Interestingly, in Cyclopamine treated animals, there was a dramatic increase in the percentage of parallel divisions (71%), when compared to control (56%). In the SAG treated animals, the percentage of parallel divisions was significantly reduced (46%) (Fig. 2R).

### Distribution of β-Catenin in anaphase cells in the EGL

**Fig. 3.**
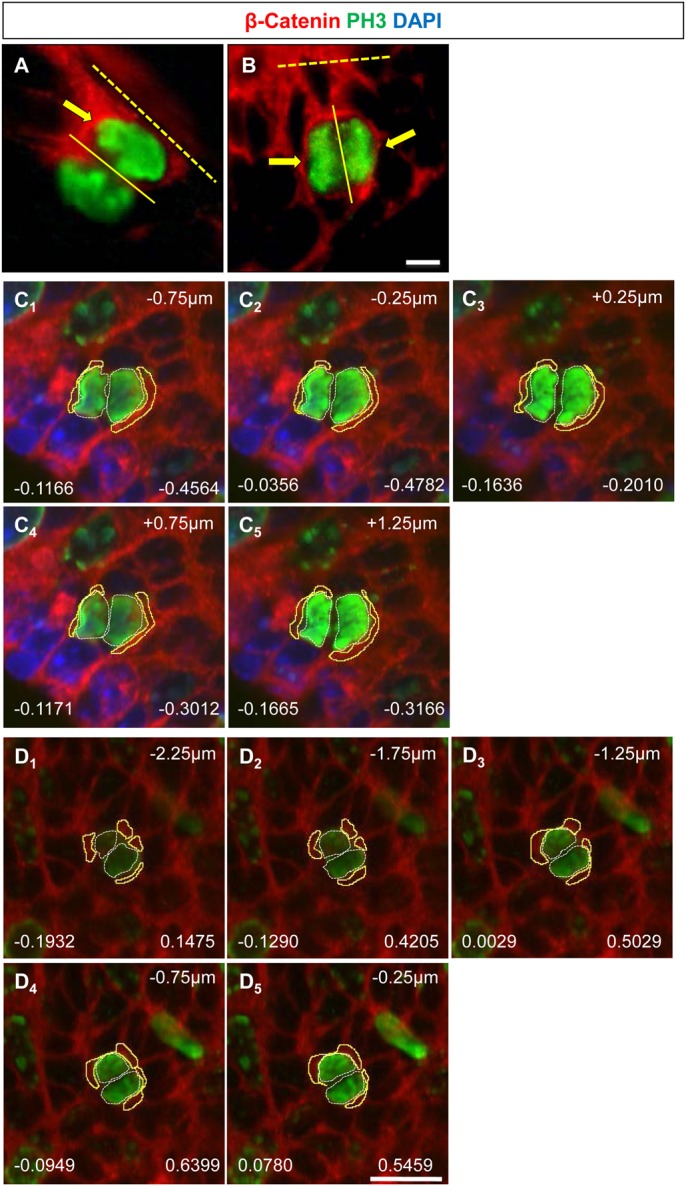
**Distribution of β-Catenin in anaphase cells of the cerebellar EGL.** (A) β-Catenin (red) is localized to the cell (yellow arrow) proximal to the pial surface (dashed line) during an asymmetric parallel division (continuous line). Cells in anaphase were identified by PH3 staining (green). (B) β-Catenin is distributed symmetrically (yellow arrows) when the plane of division (continuous line) is perpendicular to the pial surface (dashed line). (C_1-5_,D_1-5_) z-stacks through a cell dividing parallel to the pial surface. The white line around the PH3 (green) shows the ROI for which the fluorescence intensity was calculated. The value to the left shows the total intensity value for PH3 for that z-plane. The yellow line around β-Catenin (red) shows the ROI for which fluorescence intensity values for β-Catenin was calculated and this value is shown on the right for that z-plane. A negative number on the right indicates that the distal cell has a greater fluorescence intensity value and a positive number shows that the proximal cell has a greater fluorescence intensity value. Scale bar=5 µm for all the images.

The distribution of β-Catenin during GNP division was investigated (Fig. 3A,B). The ratio of fluorescence intensity of PH3 was used as a baseline since PH3 fluorescence was expected to be roughly equal between the two halves of the dividing cell and compared this distribution to that of β-Catenin. We confirmed the validity of our analysis by using a membrane marker at P6 and have compared the fluorescence intensity ratio of β-Catenin to that of the membrane marker (supplementary material Fig. S4). Two representative z-stack images are shown, with the L-R ratio for PH3 given on the left side and the L-R ratio for β-Catenin on the right side for each z-plane image (Fig. 3C1-5 and 3D1-5). Firstly, the fluorescence ratio obtained for β-Catenin was always significantly higher than for PH3 showing that it is distributed more asymmetrically than PH3 (Fig. 3C). Further, the degree of asymmetry increased significantly in P5–P9 and P10–P13 when compared to P0–P4 (Fig. 3D). The relative median values were 0.23 (lower and upper 95% CI=0.22 and 0.29) for P0–P4, 0.25 (lower and upper 95% CI=0.27 and 0.33) for P5–P9, and 0.28 (lower and upper 95% CI=0.28 and 0.36) for P10–P13. Fluorescence ratios were also plotted separately for parallel and perpendicular planes of division and this showed no particular tendency for asymmetric distribution to be associated with a specific plane of cell division. The distribution of β-Catenin was grouped into four categories: 0–0.15 (symmetric distribution), 0.15–0.30 (weak asymmetry), 0.30–0.45 (asymmetric) and >0.45 (strongly asymmetric). There is an almost two fold increase in the number of cells that are strongly asymmetric at P5–P9 and P10–P13 as compared to P0–P4 (Fig. 5A-C). In cells that were dividing asymmetrically parallel to the pial surface, at P5–P9 there was an equal probability of the proximal or the cell distal to the pial surface having more β-Catenin. However, at P0–P4 the cell distal to the pial surface was likely to have more β-Catenin and at P10–P13 it was more likely to be proximal cell (Fig. 5D). To visualize the distribution of β-Catenin asymmetry and compare it to the distribution of PH3, the percentage of cells having different fluorescence intensity values has been plotted (supplementary material Fig. S3; compare A vs. B, C vs. D, E vs. F). The frequency histograms show that the distribution of β-Catenin in GNPs changes across developmental ages and the number of cells showing highly asymmetric values increased and was present at a great frequency than that for PH3. For this analysis, all data from analyzed cells were included, even those that had a large ratio for PH3. It is possible that large asymmetric values for PH3 are due to the fact that some of the cell pairs may undergo cell death.

**Fig. 4.**
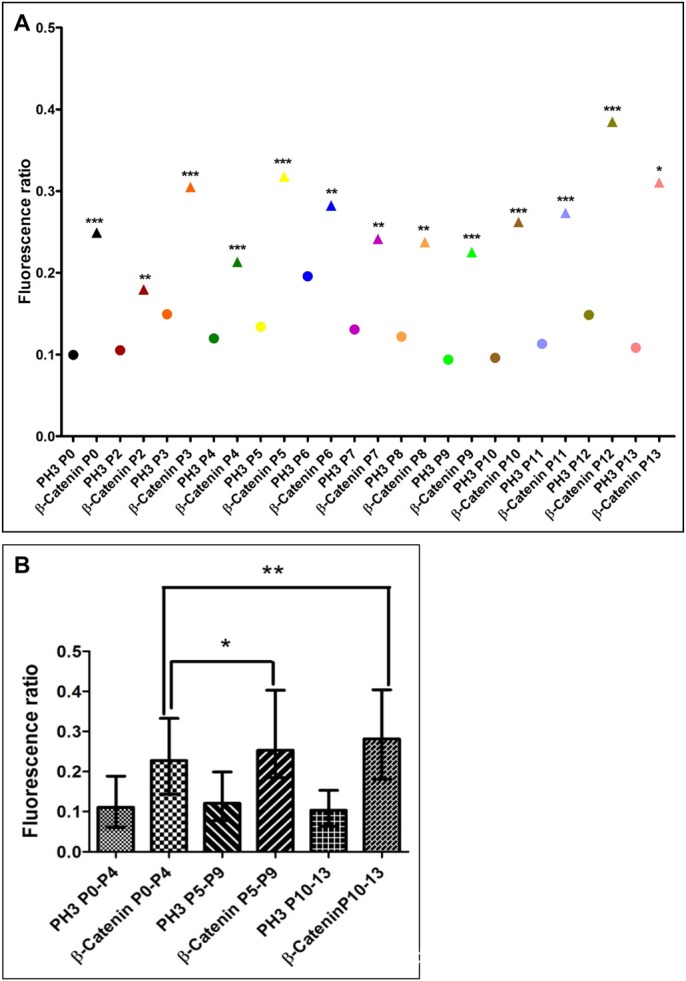
**Fluorescence intensity ratios of β-Catenin in anaphase cells of the cerebellar EGL.** (A) The graph shows the median fluorescence ratio obtained for PH3 (circle) and β-Catenin (triangle) for all the cell pairs at each age. *P* values between relative fluorescence ratio for PH3 versus β-Catenin was determined by Wilcoxon matched pair one tailed test for every cell at each age (***p<0.001, **p <0.01,*p<0.05). (B) Age grouped median fluorescence ratio with error bars representing interquartile range. The relative fluorescence ratio for β-Catenin increases significantly between P0–P4 and P5–P9 and between P0–P4 and P10–P13 (*p<0.05, **p<0.01, Mann-Whitney U test).

**Fig. 5.**
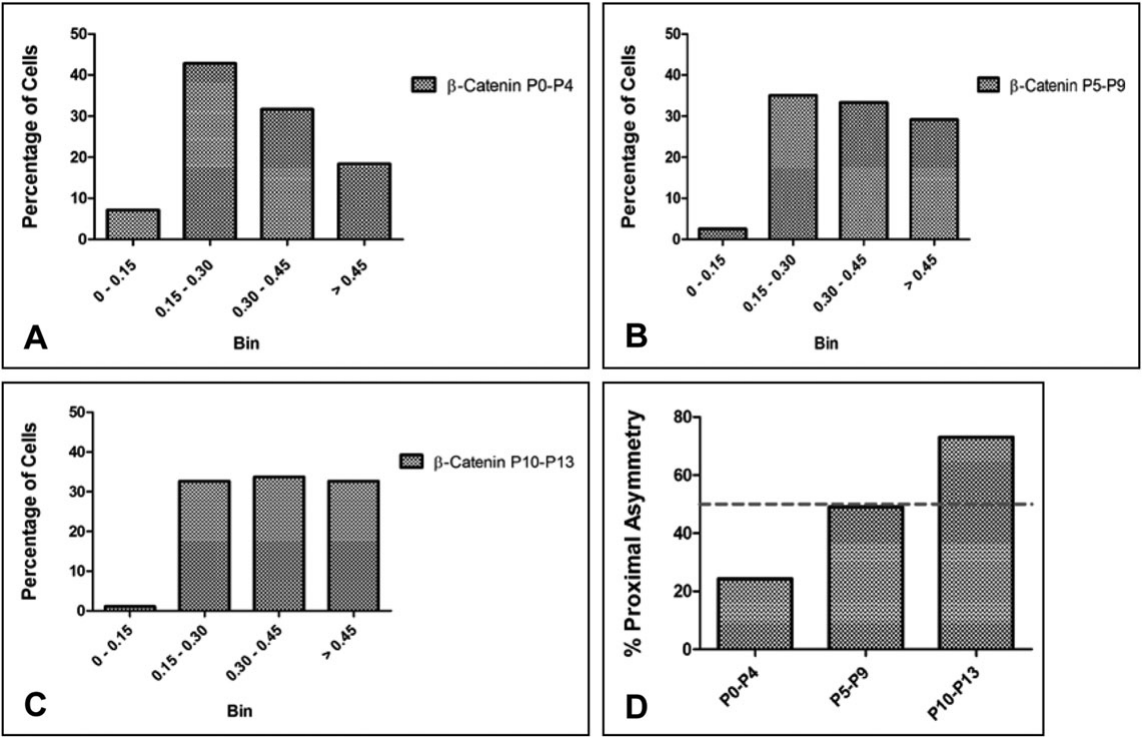
**β-Catenin asymmetry during cerebellar development.** Percentage of cells having different amounts of asymmetry: 0–0.15 (symmetric distribution), 0.15–0.30 (weak asymmetry), 0.30–0.45 (asymmetric) and >0.45 (strongly asymmetric) plotted for (A) P0–P4, (B) P5–P9 and (C) P10–P13 respectively. (D) Percentage of asymmetric cells that show more β-Catenin in the cell proximal to the pial surface at P0–P4, P5–P9 and P10–P13. The dashed line in the graph is drawn at 50% which would be the percentage of obtaining proximal asymmetry if there was no bias.

**Fig. 6.**
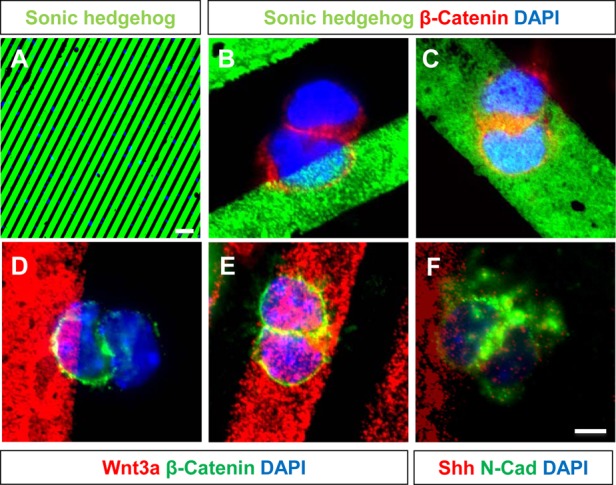
**β-Catenin is asymmetrically distributed in cells exposed to localized signalling molecules such as Shh and Wnt3a.** (A) Pattern of Shh when printed on a coverslip using PDMS stamps. (B) In a condition where one of the nuclei (blue) is in contact with the Shh stripe (green) and the other is not, β-Catenin (red) is always asymmetrically distributed to the cell that is in contact with the stripe. (C) However, when both daughter nuclei are in contact with the Shh stripe, β-Catenin is symmetrically distributed to both daughter cells. (D) When one of the daughter nuclei (blue) is in contact with the Wnt3a stripe (red) and the other is not, β-Catenin (green) is always asymmetrically distributed to the nucleus that is in contact with the stripe. (E) However, when both daughter nuclei are in contact with Wnt3a, β-Catenin is symmetrically distributed to both daughter cells. (F) N-Cadherin is asymmetrically distributed to the daughter nucleus in contact with Shh. Scale bar for A=5 µm; B-F=10 µm.

### β-Catenin is asymmetrically distributed in cells exposed to dissimilar levels of cell signalling molecules such as Shh and Wnt3a

A recent study (Habib et al., 2013) demonstrated asymmetric distribution of β-Catenin as a result of local asymmetry of signalling molecules. A similar environment was simulated using microcontact printing using both Shh and Wnt3a (Fig. 5A). Cells were seeded at low density on coverslips that were printed with Shh or Wnt3a. Cell pairs where one cell was in contact with the stripe and the other was outside the striped region were identified (20-30 cells per condition) and distribution of β-Catenin in such cell pairs were studied. It was found that β-Catenin was preferentially and asymmetrically distributed to the cell that was in contact with the Shh or Wnt3a stripe (100% of cells studied; Fig. 5B,D). When both cell nuclei were in contact with the stripe, β-Catenin was symmetrically distributed to both daughter nuclei (100% of cells studied; Fig. 5C,E). Preliminary evidence suggests that N-Cadherin is also asymmetrically distributed to the nucleus in contact with Shh (75% of cells studied; Fig. 5F).

## DISCUSSION

In this study, the orientation of dividing GNPs at different stages of postnatal cerebellar development has been characterized. Previous studies have shown that laminin that is expressed in the pia may provide a signal for outer EGL cells that strongly express the integrin alpha-6 receptor for laminin (Pons et al., 2001; Gupta et al., 2010). In addition, the mesenchyme that overlies the EGL also secretes many factors such as SDF1α that could influence GNP function (Klein et al., 2001; Reiss et al., 2002). Therefore the angle of cell division with respect to the pial surface was measured. During early postnatal cerebellum development, GNPs divide to increase the number of progenitors and it was observed that between P0 and P4 there was no significant bias in the plane of cell division. Once all the lobules were formed, the number of proliferative divisions decreased significantly and there was a corresponding increase in the number of neurogenic divisions (Corrales et al., 2006). The increase in parallel divisions at later ages observed in the current study correlates with the period of decrease in GNP proliferation during later development.

Shh controls GNP proliferation (Wechsler-Reya and Scott, 1999) and blocking Shh signalling has been shown to reduce EGL proliferation (Dahmane and Ruiz i Altaba, 1999). To establish an indirect link between levels of cell proliferation and the plane of cell division, Shh signalling *in vivo* was perturbed. Blocking Shh with a Smoothened inhibitor, Cyclopamine resulted in a reduction in cerebellar size in mice. Treatment with a Smoothened agonist, SAG resulted in an increase in surface area. These results are in agreement with previous studies that have disrupted Shh signalling (Corrales et al., 2004). Blocking of Shh signalling led to a sharp decrease in the number of perpendicular divisions and a corresponding increase in parallel divisions. Treatment with SAG brought about just the opposite – increased perpendicular divisions. As expected, there was a dramatic decrease in PCNA^+^ cells and increase in NeuroD1 population following Cyclopamine treatment. SAG treatment increased the number PCNA^+^ cells within the EGL, and lowered the number of NeuroD1^+^ positive cells. This suggests that perturbation of Shh signalling in the EGL affects the numbers of cells undergoing proliferation and differentiation and perhaps as a consequence also results in biasing mitotic spindle orientation. Whether this is a causal relationship and whether the effect is direct or indirect remains to be explored.

In the VZ of the cerebral cortex, symmetric and asymmetric distributions of proteins have been correlated with cell fate. Studies point to molecules such as Prominin-1, β-Catenin and N-Cadherin as being important cell-fate determinants (Chenn and McConnell, 1995; Kosodo et al., 2004). Notwithstanding the fact that the EGL is not directly comparable to the VZ, we wanted to check whether GNPs also show unequal distribution of proteins following cell divisions. We observed that β-Catenin was asymmetrically distributed in anaphase cells and this asymmetry became more pronounced as development proceeded. Expression of β-Catenin in the EGL showed that this asymmetry was not due to a gradient of the expression of β-Catenin in the EGL (supplementary material Fig. S1A-C). We did not address whether cells in which β-Catenin dividing symmetrically were confined to particular layers of the EGL since the characteristic of the EGL is continually changing during the first two postnatal weeks, for example the thickness of the PCNA positive or the NeuroD1 positive layers (supplementary material Fig. S2A-C). However, a study by Espinosa and Luo (Espinosa and Luo, 2008) showed that GCP divide symmetrically postnatally and GCP's that exit the cell cycle at around the same time are clonal. Whether this relates to β-Catenin distribution remains to be seen.

A recent study showed that localization of Wnt signalling by using beads to deliver the ligand in a spatially controlled manner resulted in an asymmetric distribution of β-Catenin (Habib et al., 2013). Similarly we used microcontact printing to deliver spatially restricted extracellular signals to GNPs to observe whether this resulted in a segregation of β-Catenin. It was observed that localized presence of either Shh or Wnt3a resulted in β-Catenin being asymmetrically distributed towards the ligand. Whether β-Catenin has a direct role to play in GNP cell fate remains to be seen. β-Catenin was not localized to the nucleus of all the cells in the EGL, and all cells positive for PCNA did not have nuclear β-Catenin (supplementary material Fig. S2). Further, since which of the two anaphase cells received β-Catenin was random the role of extrinsic cues in this process is not clear. This study is of particular importance because while cell fate determination in the VZ has been widely studied, the precise mechanisms that maintain the balance between GNP proliferation and differentiation are yet to be elucidated. This study has characterized the plane of division in a secondary zone of proliferation. While previous studies have linked intrinsic factors to spindle orientation, this study points to the role of extrinsic factors such as Shh that may directly or indirectly influence plane of cell division by increasing proliferation. Further analysis of the mechanisms that control mitotic spindle orientation and asymmetric distribution of molecules within the EGL and establishing a causal role of these molecules in cell fate will help in better understanding of how brain size is regulated. This in turn could help us decipher the factors contributing to disorders involving hypoplasia and overgrowth.

## MATERIALS AND METHODS

### Animals

All animal experimentation in this study was done in accordance with the guidelines laid down by the Institutional Animal Ethics Committee, of the National Brain Research Centre, and the Indian Institute of Science, India. Cerebellar tissue from C57BL/6J mice of ages P0–P14 was dissected out following hypothermia, and subsequent perfusion. These were fixed in 4% paraformaldeyde (PFA) for 24 hours and then placed in 30% sucrose. Midsagittal cryo-sections of 20–60 μm were taken. For alteration of Shh pathway in the developing mouse cerebellum, P0 animals were given a daily dose of Cyclopamine (Chen et al., 2002a) or Smoothened Agonist (SAG) (Chen et al., 2002b) for 6 days, starting at P0. Cyclopamine (ALX-430-159-M005; Alexis, Farmingdale, NY, USA) was dissolved in 100% ethanol and injected subcutaneously at 10 µg/g body weight. SAG – Smoothened agonist (ALX-270-426-M001; Alexis) was dissolved in distilled water and injected intra-peritoneally at 20 µg/g body weight. Treated animals were sacrificed at P6. Control animals were injected with the vehicle minus drug. There was no difference in the values between the two controls and hence their data was combined.

### Immunohistochemistry and Immunocytochemistry

The following primary antibodies were used in the study; β-Catenin (1:200, BD Biosciences, NC USA), Phosphohistone H3 (PH3) (1:100, Cell Signaling, Boston, MA USA), PCNA (1:100, Cell Signaling), N-Cadherin (1:200, BD Biosciences), NeuroD1 (1:100, Santa Cruz, Dallas, TX USA), β-III Tubulin (1:2000, Promega, Fitchburg, WI USA; 1:3000, Covance, Princeton, NJ USA), Shh (1:100, Santa Cruz), Wnt3a (1:100, Santa Cruz) and Cell Mask Deep Red Plasma membrane stain (1:500, Molecular Probes, Grand Island, NY USA). Alexa Fluor anti-rabbit and anti-mouse 488 and 594 secondary antibodies were used at a dilution of 1:1000 (Molecular Probes). Sections from control, cyclopamine and SAG treated mice were stained with Nissl stain. The protocols for IHC and Nissl staining have been described previously (Haldipur et al., 2012).

### Analyses

Mitotic cells were identified using anti-PH3 antibody. Midsagittal sections of 20–60 μm each were used in the study. The number of animals and cells analyzed per age is given in Table 1. The study was restricted to anaphase cells since the rotation of mitotic spindles ceases after metaphase (Haydar et al., 2003). Prediction of cleavage plane orientation in anaphase cells was performed according to (Kosodo et al., 2004). In order to ascertain the orientation of the cell with respect to the pial surface, a line was drawn between the two condensed sets of chromatin and the angle relative to the hypothetical pial plane was taken. Cell divisions were considered parallel if the plane of division was between 0 and 45 degrees. Cell divisions were considered perpendicular if the plane of division was between 45 and 90 degrees. The analysis included double-immunohistochemistry data for β-Catenin-PH3. In order to quantify the immunofluorescence of PH3 and β-Catenin in dividing GNP in the EGL of developing mouse cerebellum, we adapted the method of Bultje et al. (Bultje et al., 2009). Confocal images were taken at a thickness of 0.5–1 µm for the entire cell. A contour was drawn based on PH3 immunofluorescence, which was taken as a reference to identify the cleavage plane between the two daughter nuclei of a dividing cell at anaphase. Similarly, contours were drawn for β--Catenin surrounding the daughter nuclei. Total fluorescence intensity for PH3 and β-Catenin was calculated for all the z-sections. To confirm that we were looking at single cells we used a membrane marker and carried out the analysis at P6. The immunofluorescence quantification was performed using the formula described in Bultje et al., (Bultje et al., 2009) to get the normalized ratio for PH3 and β-Catenin. Briefly, the immunofluorescence quantification was performed using the formula:

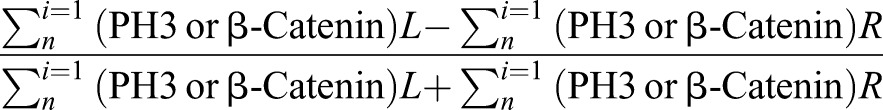

where *L* and *R* denote proximal and distal positions respectively in a parallel division and as left and right cell respectively in perpendicular division, to get the normalized ratio for PH3 and β-Catenin. The normalized ratios of PH3 and β-Catenin immunofluorescence were compared. The normalized ratios for β-Catenin and PH3 are either positive or negative. For parallel divisions, positive values denote that the cell proximal to the pial surface has more β-Catenin compared to the cell distal to the pial surface.

**Table 1.**
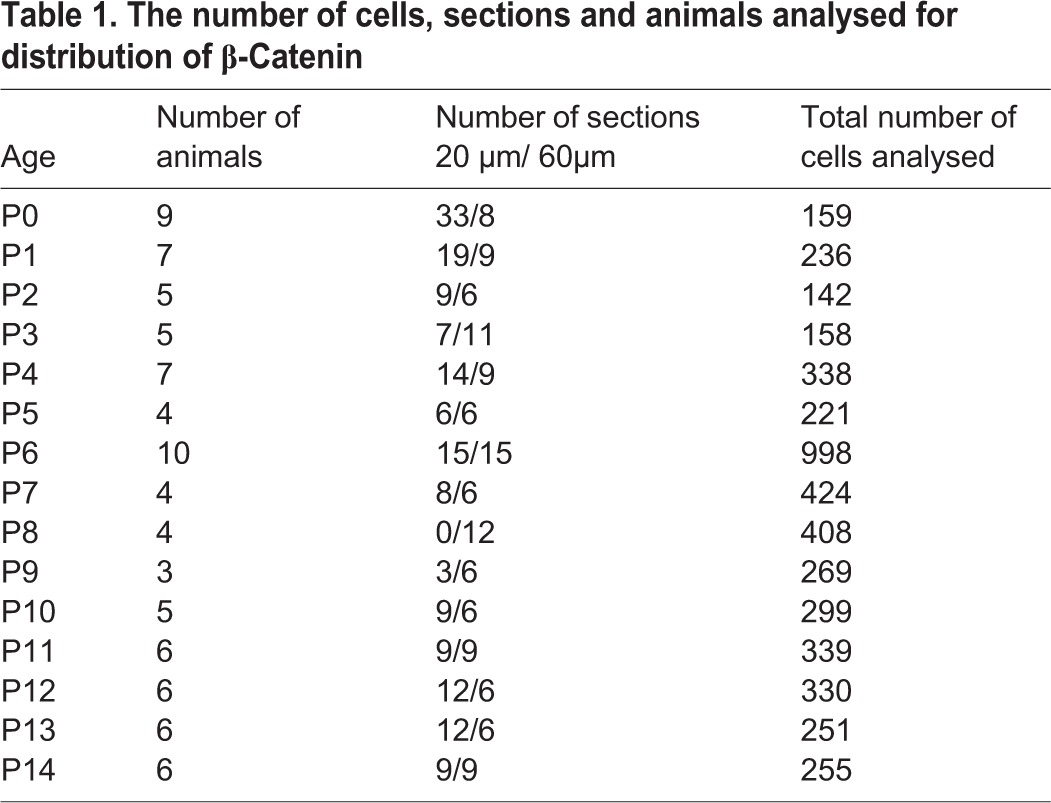
**The number of cells, sections and animals analysed for distribution of β-Catenin**

For classifying β-Catenin expression in outer and inner EGL, based on PCNA/NeuroD1 staining, the first three layers in a P6–P8 cerebellum were taken as outer EGL and the three innermost layers as the inner EGL since these layers were almost exclusively either PCNA or NeuroD1 positive respectively.

### Cerebellar granule cell culture

P6 pups were sacrificed by hypothermia; their cerebellar cortex dissected aseptically in CMF-Tyrode solution. Meninges were removed; the tissue was chopped into smaller pieces and collected in CMF-Tyrode. These were treated with trypsin-DNAse and then dissociated in the same solution by triturating to make a single cell suspension, pelleted and resuspended in serum containing media (Basal Medium Eagle, 10% horse serum, 5% fetal bovine serum and penicillin-streptomycin (Invitrogen, Grand Island, NY USA). After 12 hours, the media was replaced with serum free media [Dulbecco's modified Eagle's medium (DMEM)], B27 supplement, N2 supplement and penicillin-streptomycin (Invitrogen). Cells were seeded on Poly-D Lysine coated coverslips that were printed with Shh and Wnt3a. After 24 hours of incubation at 37°C in 5% CO2, cells were washed with 1× PBS and then fixed in 4% PFA.

### Microcontact printing

Glass coverslips (VWR, Radnor, PA USA) were cleaned and coated with Poly-D-Lysine (PDL) (0.5 mg/ml), for a minimum of 2 hours. Silicon masters were fabricated lithographically according to standard clean room protocols using SU-88 as a photoresist (von Philipsborn et al., 2006). Shh (25 µg/ml) and Wnt3a (20 µg/ml) (R&D Systems, Minneapolis, MN USA) were printed on PDL coated cover slips according to the published protocol (Mishra et al., 2008).

### Microscopy and image acquisition

All images were captured at room temperature. Apart from minor adjustment of contrast and brightness, no additional image alteration was done. All fluorescent images were captured using the Zeiss Apotome Imager M2 microscope and Zeiss Confocal LSM 510 Meta. All bright field images were captured on a Leica DFC 320.

### Statistics

The percentage of parallel divisions was calculated for different animals and weighted mean and variance for the percentages for each age was determined. Statistical difference was evaluated by unpaired *t*-test. To calculate whether there is a statistical difference between the ratio of L versus R obtained for PH3 as compared to the ratio of L versus R obtained for β-Catenin, Wilcoxon matched pair one tailed test was performed for all values obtained for each age. Mann-Whitney U test was done to test whether the L versus R ratios for β-Catenin changes with age. Statistical analyses by student *t*-test were used wherever appropriate.

## Supplementary Material

Supplementary Material
