# Supplementary material for: Asymmetric cell division of granule neuron progenitors in the external granule layer of the mouse cerebellum
Source: Biol Open. 2015 May 15;4(7):865–72. doi: 10.1242/bio.009886 (PMC4571082; doi:10.1242/bio.009886)
Supplement: Supplementary Material [file supp_4_7_865__index.html]

Asymmetric cell division of granule neuron progenitors in the external granule layer of the mouse cerebellum — Asymmetric cell division of granule neuron progenitors in the external granule layer of the mouse cerebellum — Supplementary Material 

# Asymmetric cell division of granule neuron progenitors in the external granule layer of the mouse cerebellum

## BIO009886 Supplementary Material

- Supplementary Material
