# Supplementary material for: Asymmetric cell division of granule neuron progenitors in the external granule layer of the mouse cerebellum
Source: Biol Open. 2015 May 15;4(7):865–72. doi: 10.1242/bio.009886 (PMC4571082; doi:10.1242/bio.009886)
Supplement: Supplementary Material [file supp_bio.009886_BIO009886supp.pdf]

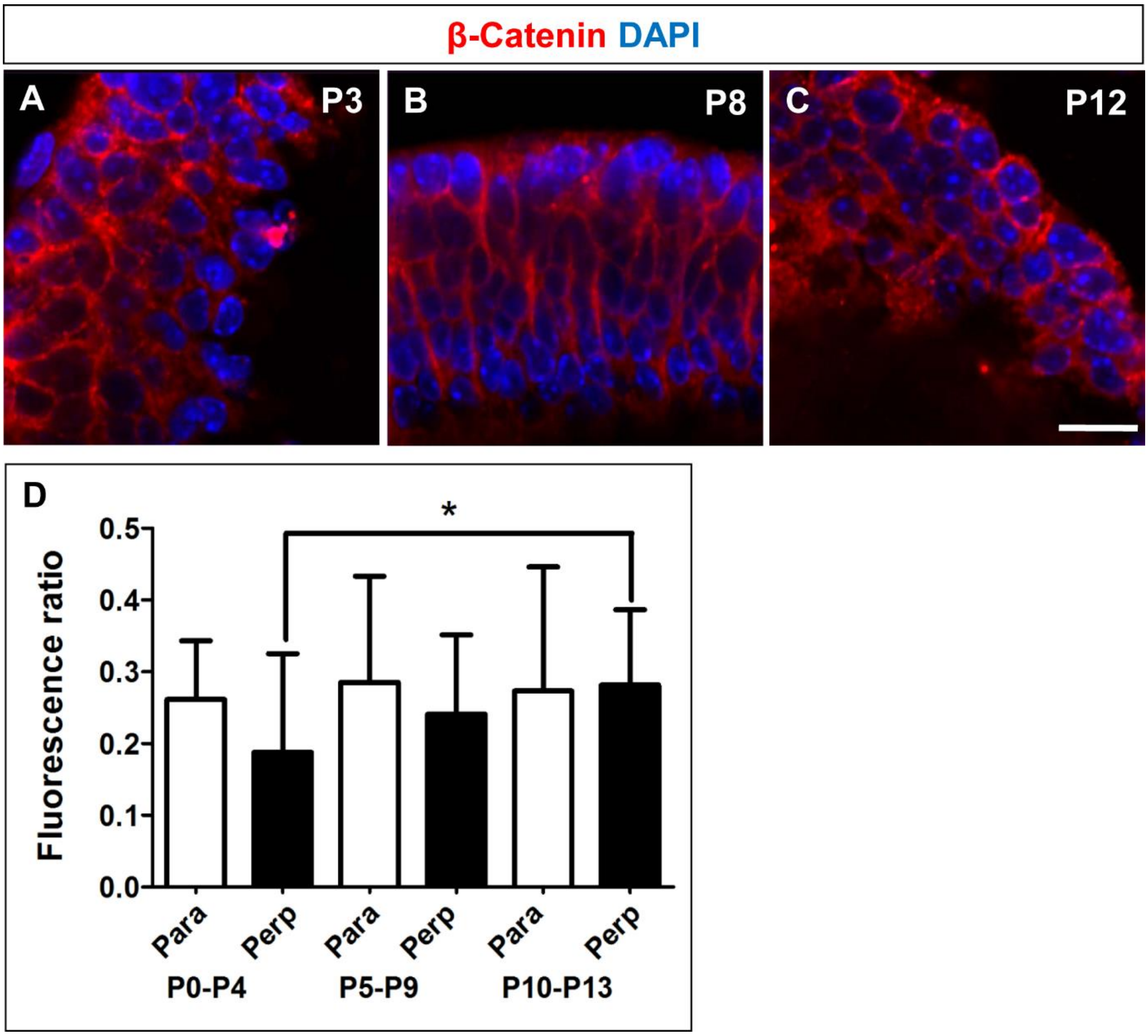

Fig. S1. Expression of β-Catenin in the EGL. (A–C) β-Catenin expression in the EGL at P3, P8 and P12 respectively. (D) Age grouped median fluorescence ratio with interquartile range for parallel (white bars) and perpendicular (black bars) planes of cell division. (*p<0.05, Mann-Whitney U test).

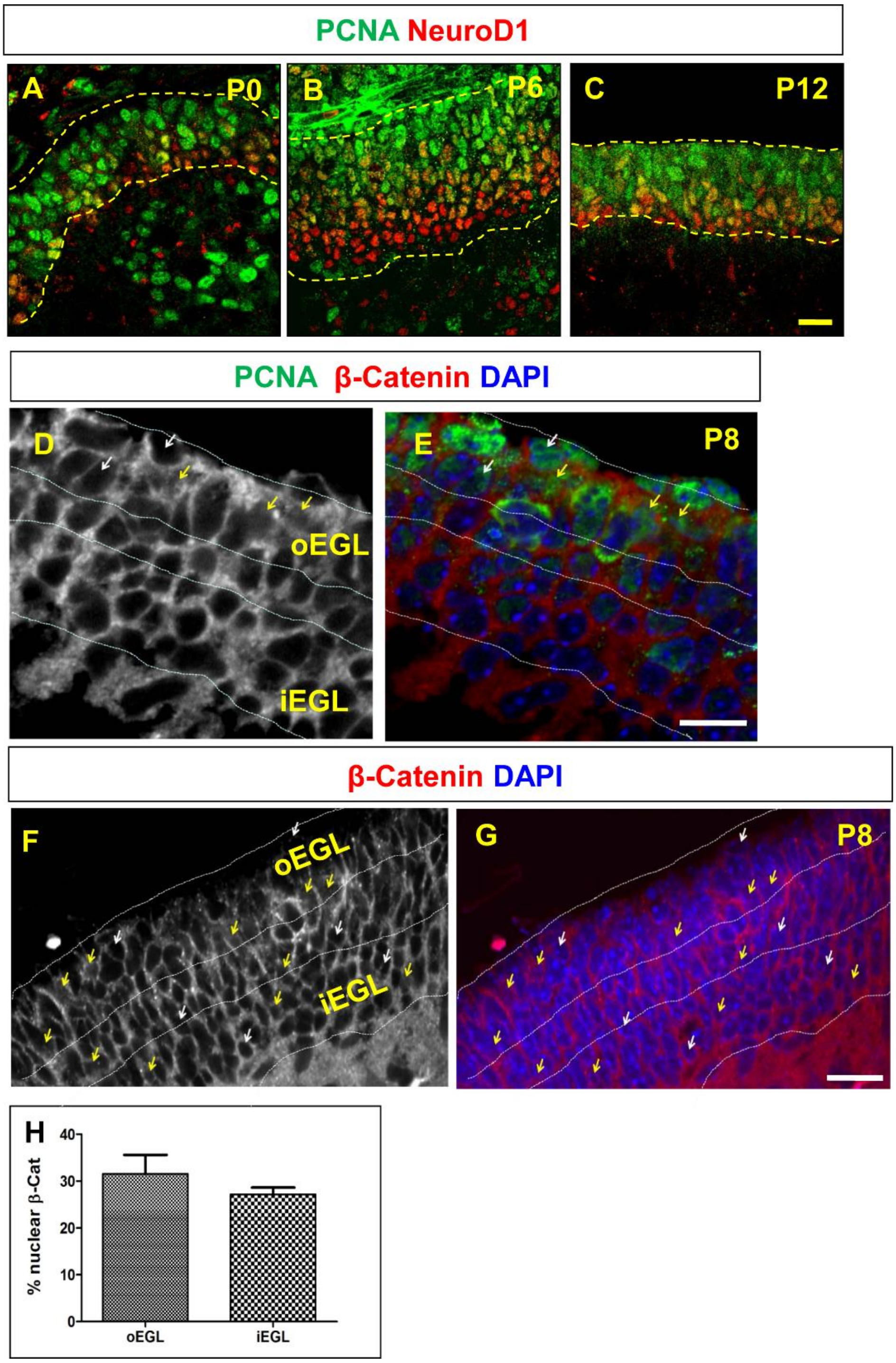

Fig. S2. Localization of β-Catenin in the EGL. (A–C) PCNA, NeuroD1 immunohistochemistry at P0, P6 and P12 respectively. (D) β-Catenin expression in grey scale showing nuclear expression (yellow arrows) and only cytoplasmic expression (white arrows). (E) β-Catenin (red), PCNA (green) and DAPI (blue). Arrows point to the same cells as in D showing that cells in the outer EGL can be PCNA positive but negative for nuclear β-Catenin. β-Catenin expression in grey scale (F) showing nuclear expression (yellow arrows) and only cytoplasmic expression (white arrows) to denote that all cells in the EGL do not have nuclear β-Catenin; β-Catenin (red) and DAPI (blue) (G). (H) The graph shows the percentage of nuclear β-Catenin in outer and inner EGL.

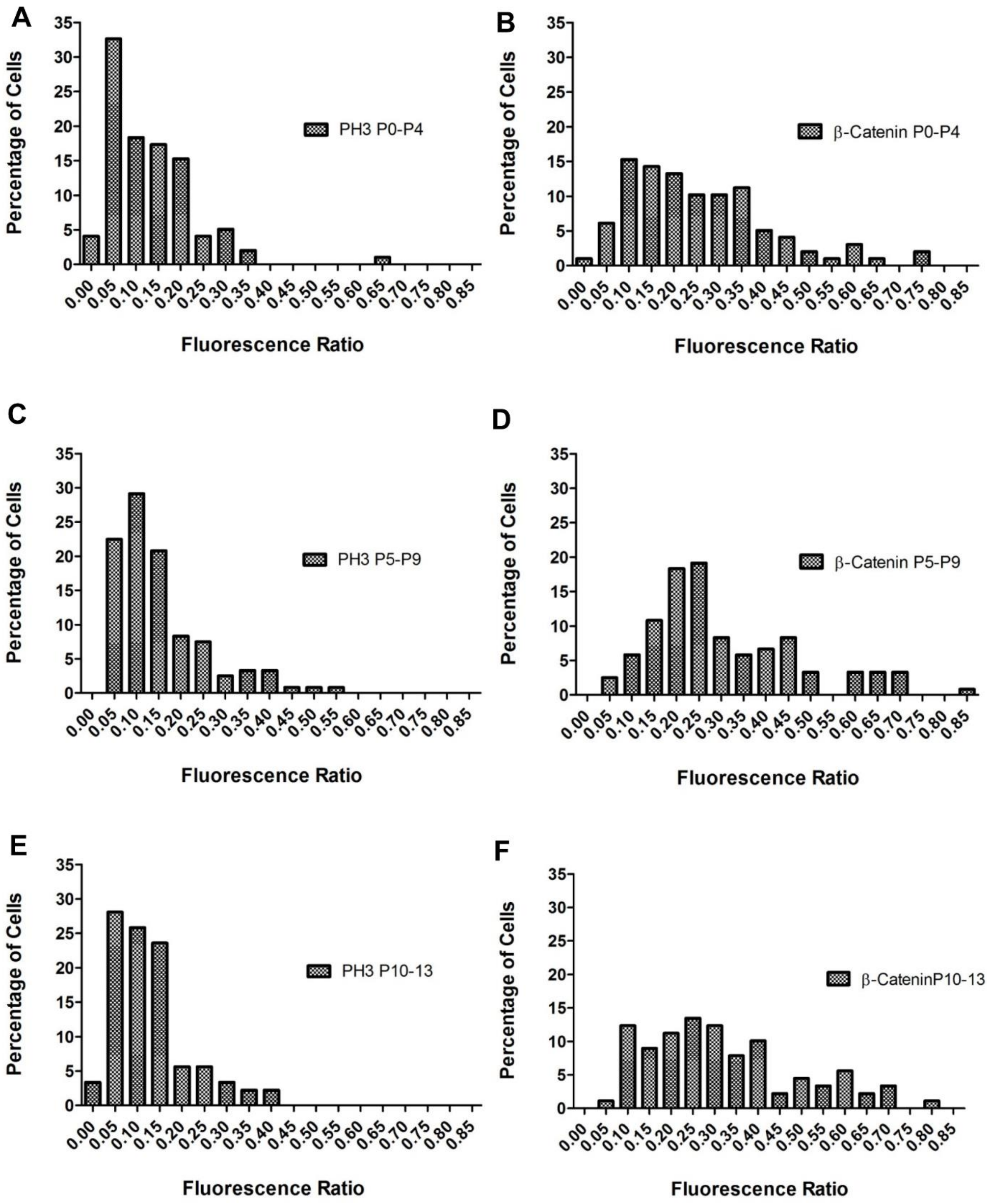

Fig. S3. Frequency histograms for PH3 and β-Catenin through cerebellar development. Frequency histograms of the relative fluorescence intensity ratio values for PH3 and β-Catenin at (A,B) P0–P4, (C,D) P5–P9, and (E,F) P10–P13 showing the percentage of cells having different fluorescence ration values (where 0 = symmetrical; 1= asymmetrical).

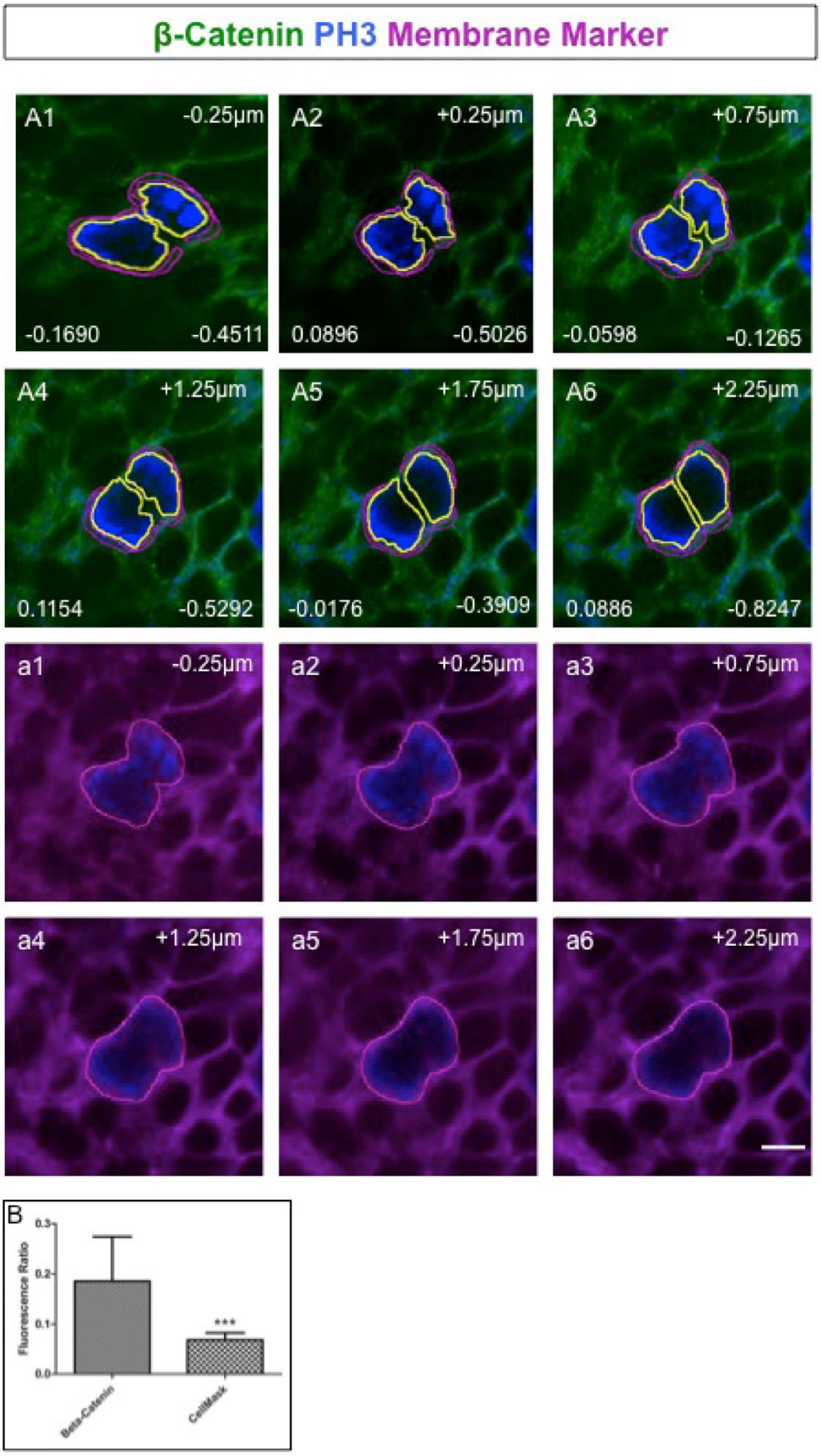

Fig. S4. Distribution of β-Catenin in P6 anaphasing cells of the cerebellar EGL with membrane marker. (A,a) z-stacks through a cell dividing parallel to the pial surface. In Figs A1–A6, the yellow

line around the PH3 (blue) shows the ROI for which the fluorescence intensity was calculated. The value to the left shows the total intensity value for PH3 for that z-plane. The magenta line around β-Catenin (green) shows the ROI for which fluorescence intensity values for β-Catenin was calculated and this is shown on the right for that z-plane. A negative number on the right indicates that the distal cell has a greater fluorescence intensity value and a positive number shows that the proximal cell has a greater fluorescence intensity value. (a1–6) the magenta line denotes the membrane boundary for that dividing cell when a membrane maker (magenta) was used. (Scale bar = 5 µm). (B) Fluorescence intensity ratios of β-Catenin and membrane marker in P6 anaphasing EGL cells. The histograms represent the median fluorescence ratio with interquartile range. P values between relative fluorescence ratio for β-Catenin versus membrane marker was determined by Wilcoxon matched pair one tailed test (***p<0.001).
